# Utilization of psychotropic medications in individuals with autism spectrum disorder

**DOI:** 10.1186/s12888-025-07714-2

**Published:** 2025-12-23

**Authors:** Paula A. Jaimes-Buitron, Leslie Neely, Melissa Dziuk Svoboda, Gretchen Gemeinhardt, Carolina Vivas-Valencia

**Affiliations:** 1 Department of Biomedical Engineering and Chemical Engineering, The University of Texas at San Antonio, San Antonio, TX USA; 2 Department of Educational Psychology, The University of Texas at San Antonio, San Antonio, TX USA; 3 Baylor College of Medicine, The Children’s Hospital of San Antonio, San Antonio, TX USA; 4 UT Health School of Public Health, Houston, TX USA

**Keywords:** Autism spectrum disorder, Psychotropic medications, Medication switching, Prescribing changes, Behavioral problems, ASD treatment

## Abstract

**Background:**

Second-generation antipsychotics (SGAs), specifically aripiprazole and risperidone, and selective serotonin reuptake inhibitors (SSRIs), are psychotropic medications commonly prescribed to individuals with autism spectrum disorder (ASD). However, it is unclear how often individuals with ASD initiate with SGAs, how frequently they switch medications, and how utilization has changed over time. This study investigates trends in psychotropic medication use among individuals with ASD in the USA from 2012 to 2021.

**Methods:**

We analyzed a national cohort of commercially insured individuals with ASD aged 2 to 26 years using data from the IQVIA PharMetrics Plus for Academics database. Individuals were classified as newly prescribed if they had not previously received a prescription for the medication before the study period, and no prescription in the period immediately preceding it. We examined trends in psychotropic medication utilization yearly and conducted a detailed month-by-month analysis for 2019. A Mann-Whitney U test was used to compare medication switching rates between SSRIs and SGAs.

**Results:**

Among 24,730 individuals, 64.6% were prescribed SGAs or SSRIs. Medication switching was more frequent among those initially prescribed aripiprazole or risperidone (6.13% annually) compared to those starting with SSRIs (3.41%). The Mann-Whitney U test (W = 67, *p* < 0.05) confirmed a significant difference in switching rates between the two groups. Switching from risperidone decreased from 2012 to 2021 (Spearman’s ρ = -0.32), whereas switching from aripiprazole increased (Spearman’s ρ = 0.50).

**Conclusions:**

Individuals with ASD newly prescribed SGAs switched to other drug classes nearly twice as often as those prescribed SSRIs. The higher switching rate may be influenced by adverse effects or insufficient symptom improvement. Future studies should explore long-term outcomes and the clinical decision-making processes underlying medication changes.

**Clinical trial number:**

Not applicable.

**Supplementary Information:**

The online version contains supplementary material available at 10.1186/s12888-025-07714-2.

## Background

Autism spectrum disorder (ASD) is a neurodevelopmental condition for which diagnostic rates in the United States have increased from 6.7 per 1,000 children in 2000 to 32.2 per 1,000 children in 2022 [1]. This increase is in part due to heightened awareness, broadened diagnostic criteria, and advancements in screening practices [2, 3]. ASD is characterized by differences in social communication and engagement in restricted and repetitive behaviors [4]. Additionally, individuals with ASD often exhibit co-occurring conditions, including behaviors such as aggression and self-injury [5]. In fact, research indicates that up to 50% of individuals with ASD experience some level of aggression [6], while 42% engage in self-injurious behaviors [7]. Interventions, such as psychotropic medications and therapy based on the science of behavior analysis, are the front-line interventions to help manage associated behaviors and improve daily functioning [8, 9].

Recent studies report that 50–64% of children and adolescents with ASD and 59–79% of adults with ASD are prescribed psychotropic medications [9–12]. These medications are often prescribed to address issues related to behavioral functioning and mood in individuals with ASD [13–15]. While there are no FDA-approved medications that specifically target the core symptoms of ASD, two second-generation antipsychotics (SGAs) (risperidone and aripiprazole) are FDA-approved to treat irritability and aggression in individuals with ASD [15, 16]. Other off-label drug classes have been used to treat behaviors in individuals with ASD, such as selective serotonin reuptake inhibitors (SSRIs) [15, 17, 18].

While SGAs such as risperidone and aripiprazole have shown clinical effectiveness for managing irritability and aggression in individuals with ASD, they are associated with metabolic complications, weight gain, and sedation [15, 19, 20]. SSRIs are generally better tolerated, but their efficacy in ASD-related behavioral symptoms is limited, and some studies report the emergence of behavioral activation (e.g., irritability or aggression) [21–24]. This variability in efficacy and side effect profiles, coupled with the absence of standardized treatment guidelines, may contribute to widespread polypharmacy and frequent medication changes [5, 25]. For example, recent research by Feroe et al. (2021) revealed frequent changes in medication regimens among individuals with ASD, with high rates of polypharmacy and substantial variability in prescriptions even within the same drug class. These results highlight the complexity and inconsistency of pharmacological management in this population. Building on these findings, this study utilizes a multi-year, nationally representative cohort that includes prescription records to quantify how frequently individuals with ASD initiate treatment on SGAs versus SSRIs, the rates at which they switch medications, and how these changes have evolved from 2012 to 2021. We hypothesize that individuals with ASD are more likely to initiate treatment with SGAs because these medications are FDA-approved for managing behavioral symptoms in this population. Additionally, as the incidence of ASD has increased over time, we expect an overall rise in the utilization of psychotropic medication, which may also lead to higher observed switching rates across time. Finally, we hypothesize that individuals with ASD will demonstrate higher switching rates when treated with SGAs compared to SSRIs. Ultimately, the results of this study may provide a better understanding of how psychotropic medications are used in this population and contribute real-world evidence to support future efforts in knowledge discovery and medication management.

## Methods

### Study data and population

This study followed the STROBE reporting guidelines, a set of recommendations designed to improve the reporting quality of observational studies in epidemiology [26]. It was exempt from review by the University of Texas at San Antonio Institutional Review Board due to de-identified preexisting data. We analyzed a sample from the IQVIA PharMetrics Plus for Academics database of primarily commercially insured individuals to identify individuals with at least two claims on different dates containing diagnostic codes for ASD based on the International Classification of Diseases, Ninth Revision (ICD-9) and International Statistical Classification of Diseases and Related Health Problems, Tenth Revision (ICD-10) (ICD-9 codes 299.0, 299.00, 299.01, 299.8, 299.80, 299.81, 299.9, 299.90, 299.91, 299, 299.1, 299.10, and 299.11; ICD-10 codes F84, F84.0, F84.3, F84.5, F84.9, and F84.8) [10]. This approach follows a validated method to identify individuals with ASD using claims datasets, and has been shown to generate a predictive accuracy of 90% [27]. We included individuals aged 2 to 26 with at least 12 months of continuous enrollment from January 2012 to December 2021. The study period was restricted to 2012–2021 to ensure a more robust and representative sample. Before 2012, the number of recorded ASD cases in the database was limited, which could compromise the reliability of trend analyses. Data from 2022 was also excluded due to incomplete records for the full calendar year.

### Measurements

#### Medication initiation

**Fig. 1:**
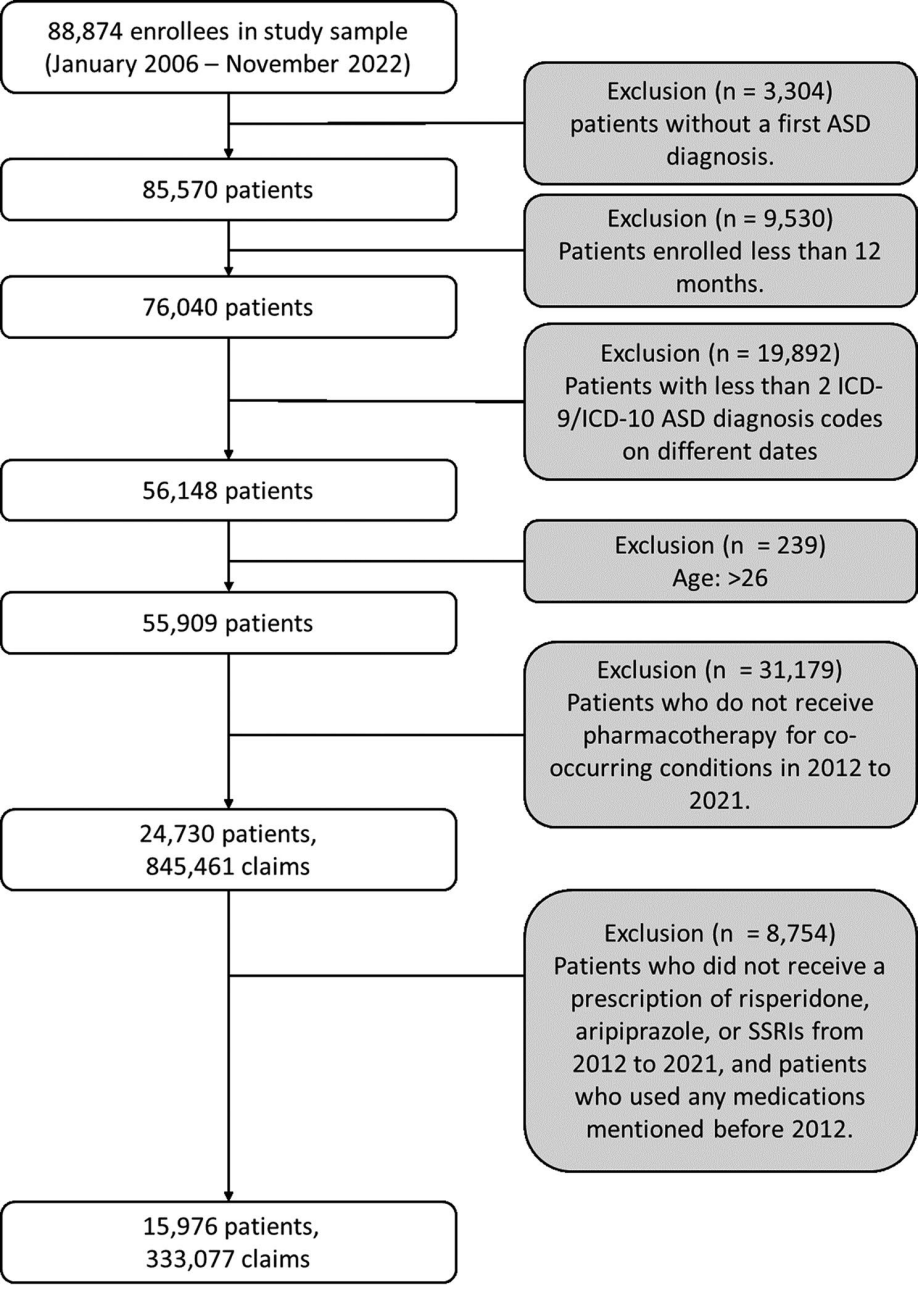
Flowchart of individuals included in the study

We clustered pharmaceutical information into two drug classes: SSRIs and SGAs. For this study, SGAs were limited to the FDA-approved medications risperidone and aripiprazole. We also identified individuals who were newly prescribed SSRIs (including fluoxetine, fluvoxamine, sertraline, citalopram, escitalopram, and paroxetine), which were treated as a single class encompassing all SSRIs included in the dataset, to assess differences in medication changes between drug classes for the two groups (See Fig. 1). This comparison provides insights into whether utilization and switching behaviors differ by therapeutic class. In this study, the term “newly prescribed” refers to individuals with no recorded use of the medications before their first prescription within the study period. To confirm their status as new users, we verified that there was no record of prior prescriptions for the specified medications (risperidone, aripiprazole, or SSRIs) from 2006 to 2012 in the dataset. An individual was considered a new user in a specific year if they initiated or reinitiated the medication that year and had not been prescribed it in the preceding year. For instance, an individual prescribed aripiprazole for the first time in 2014, with no previous use of the medication between the beginning of our available data (2006) to the index year (2012), would be classified as a new user for that year and as a new user overall. Conversely, an individual who starts an SSRI in 2015 after not using it in 2014 would be a new user for 2015, but not a new user overall, if they had used the same SSRI at any time between 2006 and 2014. This approach accounts for both pre-2012 usage and year-to-year initiation changes.

It is important to note that individuals could have previously received other medications outside of the selected drug classes. For example, an individual newly prescribed aripiprazole could have previously used an alpha-2 agonist; such transitions are captured in the medication switching analyses described below. We emphasize that our measure reflects class-level treatment changes rather than clinical discontinuation per se. This distinction clarifies that we are assessing patterns of switching between psychotropic drug classes and not inferring the clinical rationale for stopping or continuing a specific medication.

#### Medication switching

We defined medication switching as a change in psychotropic drug class, specifically from an SSRI to an SGA or vice versa, between two consecutive time periods (either years or months). Initial analyses of switching were conducted at the class level, with SGAs (limited to risperidone and aripiprazole) treated as a single group and SSRIs treated as a class. To qualify as a switch, individuals had to be prescribed only one psychotropic class during the baseline period and a different one in the subsequent period, with no overlapping prescriptions. This criterion was applied to isolate intentional or clinically guided changes in treatment class, rather than concurrent combination therapy. Switches were assessed at both annual (2012 to 2021) and monthly intervals for 2019 to capture short- and long-term prescribing changes. We also tracked the direction of switching (e.g., from SSRI to SGA versus from SGA to SSRI).

#### Analysis

We analyzed data across the entire study period to examine longitudinal trends in the utilization of newly prescribed psychotropic medication. We conducted a detailed month-by-month analysis for 2019, chosen for the highest number of prescriptions and individuals within a single year. This made 2019 a robust reference point for studying short-term prescription and medication changes. We generated a Sankey Diagram [28] only including individuals prescribed a single medication each year or month, making the bars mutually exclusive. Medications were aggregated by the targeted drug classes. The height of each vertical bar represents the number of individuals prescribed a given medication in a specific year. The connecting lines between bars indicate whether individuals continued with the same medication or switched to a different one in the following year or month. The thickness of these lines reflects the proportion of individuals who remained on the same medication versus those who switched to another. Individuals with more than one medication per year are not represented.

To compare medication switching rates between SSRIs and SGAs, we conducted a Mann-Whitney U test [29], using class-level switching. This non-parametric test is ideal for evaluating two independent distributions when we cannot assume normality. It helps us determine whether the switching rates differ between the two groups without the requirement of equal variance. In this analysis, the W value represents the sum of ranks for one group, after all data points from both groups are combined and ranked from lowest to highest. Specifically, Group A comprises individuals switching from SGAs (limited to risperidone and aripiprazole) to other medications, and Group B includes individuals switching from SSRIs to other medications on an annual basis. To further examine trends in medication switching from 2012 to 2021, we used Spearman’s rank correlation [30], considering SGAs separately (risperidone and aripiprazole) versus SSRIs, allowing us to examine differences in switching at the individual drug level. Positive values indicate an increase in switching behavior and negative values indicate a decrease in switching behavior over time.

## Results

Among the 24,730 individuals included, 15,976 (64.60%) were treated with SGAs or SSRIs. The majority of prescriptions were for males (11,805, 73.90%). The most common age at first ASD diagnosis was between 12 and 17 years (6,028, 37.70%). In comparison, individuals in the same database who did not receive any psychotropic medications were younger at their first ASD claim and had a higher proportion of early childhood diagnoses (ages 2–5) (Supplementary Table S1), highlighting differences between medicated and non-medicated cohorts. In terms of drug class prescriptions, SSRIs were prescribed to 58.76% (9,387) of individuals, while 16.66% (2,662) received SGAs (limited to risperidone and aripiprazole), and 24.58% (3,927) received both classes. Both classes have a similar episode duration (prescriptions of the same medication with a gap of less than 30 days), with SSRIs episodes lasting slightly longer (226.86 days vs. 221.86 days). During the study period, the year 2019 saw the highest peak of individuals with ASD receiving psychotropic medications (7,469, 46.75%) (See Table 1).

**Table 1:** Demographic characteristics and number of individuals by calendar years between 2012 and 2021^a^

Characteristic	No. of participants (%)			
	All (*n* = 15,976)^b^	Only SGAs (*n* = 2,662)^d^	Only SSRIs (*n* = 9,387)^e^	Both SGAs and SSRIs (*n* = 3,927)^c^
Sex				
Female	4,171 (26.10)	506 (19.00)	2,725 (29.00)	940 (23.90)
Male	11,805 (73.90)	2,156 (81.00)	6,662 (71.00)	2,987 (76.10)
Age at first ASD diagnosis				
2 to 5	1,136 (7.10)	280 (10.50)	550 (5.90)	306 (7.80)
6 to 11	5,033 (31.50)	908 (34.10)	2,770 (29.50)	1,355 (34.50)
12 to 17	6,028 (37.70)	847 (31.80)	3,705 (39.50)	1,476 (37.60)
18 to 26	3,779 (23.70)	627 (23.60)	2,362 (25.20)	790 (20.10)
Calendar year				
2012	1,707	507 (29.70)	924 (54.13)	276 (16.17)
2013	2,292	610 (26.61)	1,313 (57.29)	369 (16.10)
2014	2,597	599 (23.07)	1,577 (60.72)	421 (16.21)
2015	2,971	699 (23.53)	1,797 (60.48)	475 (15.99)
2016	3,619	795 (21.97)	2,313 (63.91)	511 (14.12)
2017	4,619	953 (20.63)	2,998 (64.91)	668 (14.46)
2018	5,943	1,286 (21.64)	3,638 (61.21)	1,019 (17.15)
2019	7,469	1,541 (20.63)	4,678 (62.63)	1,250 (16.74)
2020	6,838	1,261 (18.44)	4,518 (66.07)	1,059 (15.49)
2021	6,970	1,217 (17.46)	4,678 (67.12)	1,075 (15.42)
Episode duration in days (Mean (SD))^f^	230.91 (314.44)	221.86 (301.93)	226.86 (305.73)	237.94 (327.21)

Abbreviations: ASD, autism spectrum disorder. SSRIs, selective serotonin reuptake inhibitors. SGAs, second-generation antipsychotics

^a^ The estimations of each demographic variable are calculated from the individuals who received a prescription of any of the two SGA medications (aripiprazole and risperidone) and/or SSRIs. Individuals included in the estimations can also receive medications different from those mentioned.

^b^ “All” is the total estimation of individuals with at least one prescription of SSRIs or SGA medications (limited to aripiprazole and risperidone)

^c^ “Both SGAs and SSRIs” indicates the individuals receiving the two drug classifications in the same year without verification of concurrency

^d^ “Only SGAs” are the individuals who received aripiprazole or risperidone, the two SGAs selected

^e^ “Only SSRIs” are the individuals who received only medications from the specified category, including fluoxetine, fluvoxamine, sertraline, citalopram, escitalopram, and paroxetine

^f^ Episodes were defined as a set of prescriptions for the same medication with a gap of less than 30 days between them

**Fig. 2:**
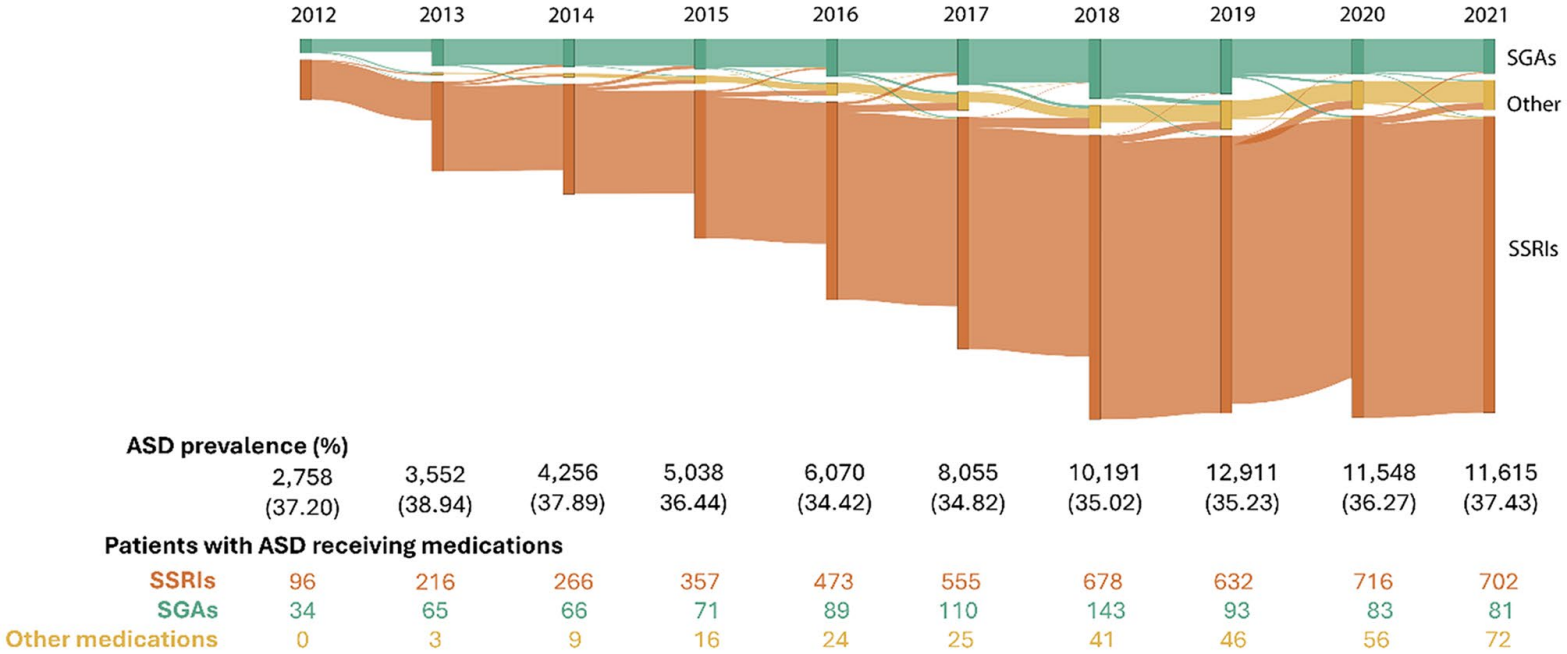
Annual trends and changes in the utilization of medications. Legend: Each vertical bar represents the number of individuals prescribed a single medication class in a given year. The connecting lines indicate whether individuals continued the same medication or switched to another class the following year. For more details on diagram construction and inclusion criteria, see the Methods section. The “SGAs” stand for “second-generation antipsychotics” and includes aripiprazole and risperidone. “SSRIs” are selective serotonin reuptake inhibitors, including fluoxetine, fluvoxamine, sertraline, citalopram, escitalopram, and paroxetine, and the “Other” classification corresponds to medications belonging to different drug classes than those selected for the study. The table shows the proportion of individuals with ASD who received at least one psychotropic medication in each calendar year. The volume of individuals per drug class, which represents the individuals who received any of those medications by year, is indicated by the size of the vertical bars

We found that individuals who were prescribed SGAs first had higher observed switching rates between drug classes than those who were given SSRIs first. Specifically, 6.13% (2.94% aripiprazole, 3.19% risperidone) of individuals starting with SGAs changed to different drug classes between years, as compared to 3.41% of individuals who began with SSRIs (see Fig. 2). Furthermore, switching from SGAs to other drug classes occurred more frequently than switching from SSRIs yearly (W = 67, p-value < 0.05). The switch from 2012 to 2013 recorded the highest shift (11.76%) from SGAs compared to SSRIs. See Supplementary Figures S2-S3 for details regarding the individual count for risperidone and aripiprazole.

**Fig. 3:**
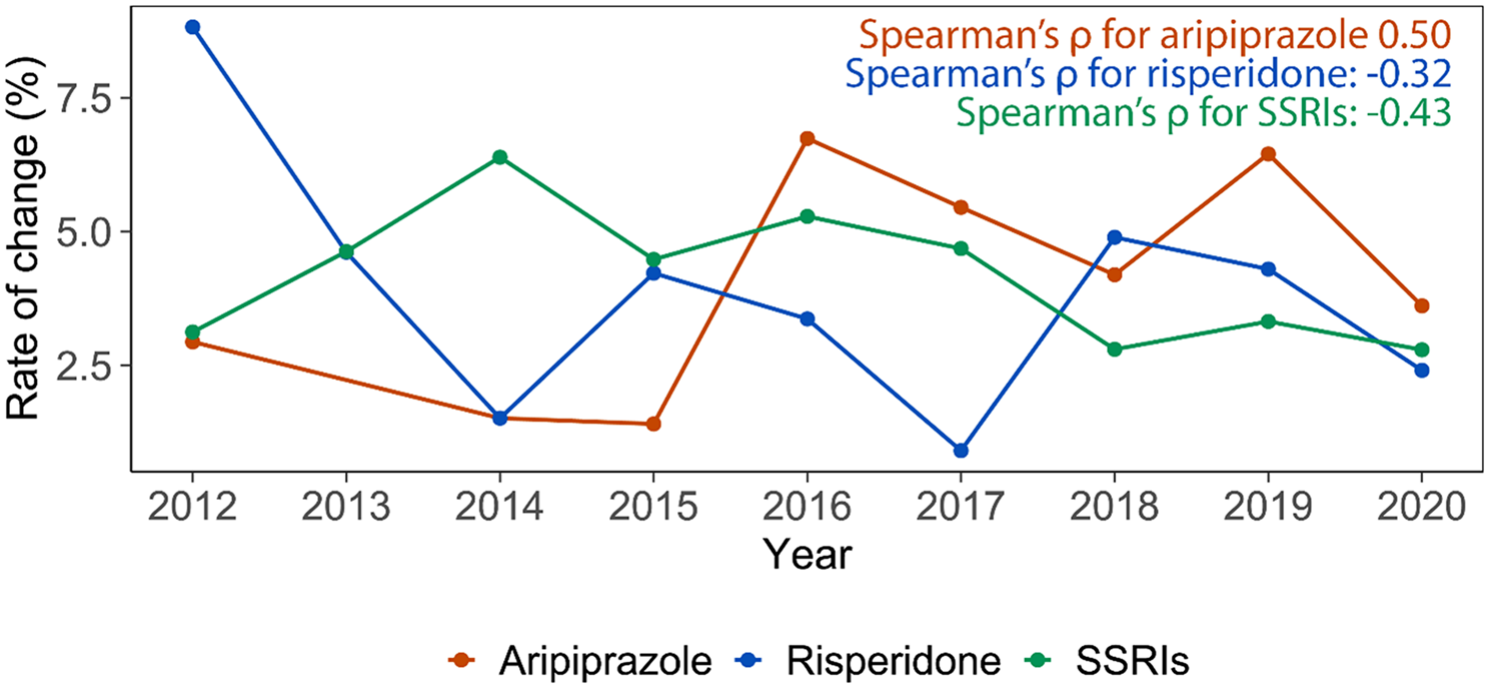
Proportion of individuals switching from aripiprazole, risperidone, or SSRIs to other medications yearly. Legend: Rate of change represents the proportion of individuals who started using aripiprazole or risperidone (SGAs), or SSRIs (including fluoxetine, fluvoxamine, sertraline, citalopram, escitalopram, or paroxetine) by year and switched to other medications. The proportion was calculated by dividing the number of individuals who switched by the total number of individuals who started using any of those medications yearly. The “Spearman’s $\:{\uprho\:}$” means Spearman’s rank correlation coefficient. A positive correlation coefficient indicates that the rate of change for a medication increases over time, while a negative correlation coefficient means the rate of change decreases over time

**Fig. 4:**
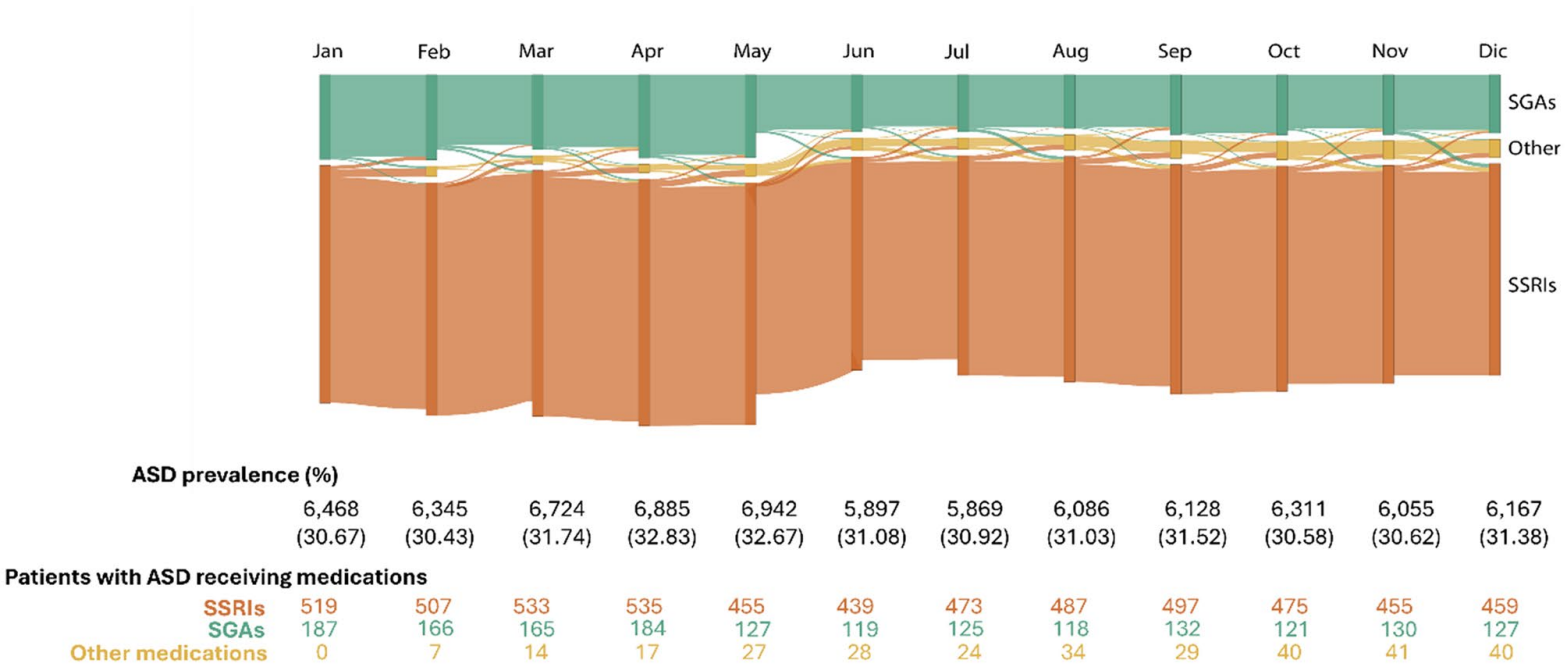
Monthly trends and changes in the utilization of medications – 2019. Legend: Each vertical bar represents the number of individuals prescribed a single medication class in a given month. The connecting lines indicate whether individuals continued the same medication or switched to another class the following month. For more details on diagram construction and inclusion criteria, see the Methods section. The “SGAs” stand for “second-generation antipsychotics” and include aripiprazole and risperidone. “SSRIs” are selective serotonin reuptake inhibitors, including fluoxetine, fluvoxamine, sertraline, citalopram, escitalopram, and paroxetine, and the “Other” classification corresponds to medications belonging to different drug classes than those selected for the study. The table shows the proportion of individuals with ASD who received at least one psychotropic medication in each month. The volume of individuals per drug class, which represents the individuals who received any of those medications by month, is indicated by the size of the vertical bars

Additionally, the analysis revealed distinct trends in medication use over time for risperidone and aripiprazole. For risperidone, switching to other drug classes became less frequent over the years (Spearman’s $\:{\uprho\:}$ = -0.32; see Fig. 3). By contrast, switching from aripiprazole to other drug classes increased over time (Spearman’s $\:\rho\:$ = 0.50). This highlights a notable divergence in prescribing changes between the two SGA medications. For example, monthly switching in 2019 showed that 4.89% of SGA users switched to other drug classes (2.51% aripiprazole, 2.38% risperidone), and 3.01% switched from SSRIs (see Fig. 4). In addition, switching between the two SGAs were relatively rare, with 1.23% of individuals switching from risperidone to aripiprazole and 1.44% switching from aripiprazole to risperidone annually, and 0.48% of individuals switching from risperidone to aripiprazole on a monthly basis in 2019; no individuals switched from aripiprazole to risperidone monthly. See Supplementary Figures S2-S3 for details regarding the individual count for risperidone and aripiprazole.

## Discussion

This study examined psychotropic medication use among individuals with ASD in the U.S. from 2012 to 2021 using claims data. Notably, individuals newly prescribed SGAs were nearly twice as likely to switch to a different medication class compared to those newly prescribed SSRIs. Furthermore, risperidone and aripiprazole showed different trends, with switching from risperidone declining over time, while switching from aripiprazole increased. Although the overall switching rates between the two SGAs are low, the rate of individuals who began treatment with aripiprazole and then switched to risperidone is higher than the rate of those who started with risperidone and switched to aripiprazole. These differences may reflect variations in efficacy and tolerability: risperidone is generally considered more effective for managing behavioral symptoms, but has a higher risk of metabolic side effects [31], whereas aripiprazole is often better tolerated but may require closer clinical monitoring, especially when used as long-term treatment with high doses [31–34]. Short-term adjustments and careful monitoring during switching are consistent with standard clinical practice in managing SGA therapy [35], and some individuals may initially try another medication within the same drug class before moving to a different class [10]. These findings suggest switching profiles in the utilization of psychotropic medications and highlight potentially important differences in medication tolerability and treatment response. These temporal patterns may also have been influenced by the availability of generic formulations, with risperidone becoming generic in 2008 and aripiprazole in 2015, potentially increasing accessibility and affecting prescribing strategies [36].

It is important to note that most of the individuals in our cohort received their first ASD diagnosis between 12–17 years (37.70%), with individuals receiving SGAs being diagnosed at 6–11 years (34.10%) and those receiving SSRIs being diagnosed at 12–17 years (37.60%). These findings may reflect the timing of recognition of behavioral problems, which studies suggest tends to peak between the ages of 15 to 34, although such problems can emerge at any age [15, 37]. Notably, SGAs such as risperidone and aripiprazole are approved for the treatment of behavioral problems, including irritability and aggression, in children with ASD. Risperidone is approved for children aged 5 to 16 years [17]. In contrast, SSRIs should be avoided in children with ASD due to their limited effectiveness and a higher risk of side effects [23, 24]. It is important to note that the first diagnosis captured in the dataset may not reflect the true age at clinical diagnosis, since information prior to 2006 is not available in the database.

While the exact reasons for the medication switching cannot be determined from claims data, differences in efficacy, tolerability, and clinical management likely contribute to the switching pattern observed in this study [13–15]. SSRIs are generally well tolerated but show limited efficacy for irritability or aggression in children and adolescents with ASD, and some trials report the emergence of behavioral problems or related side effects [21–24]. In contrast, FDA-approved SGAs have demonstrated effectiveness in reducing irritability, aggression, and other disruptive behaviors [15, 19, 20]; however, these benefits must be weighed against their side effects, particularly metabolic risks associated with risperidone and other SGAs [9, 17, 38]. For some individuals, the therapeutic benefits of SGAs may initially outweigh these risks, which could explain the early use of these medications and subsequent adjustments as clinicians may balance symptom control with emergent side effects [8, 17, 19, 34, 39].

The differences between risperidone and aripiprazole may further help explain the variations in switching patterns. Risperidone is often regarded as highly effective for managing behavioral symptoms but carries a greater risk of metabolic complications, which may prompt transitions to other SGAs for individuals who experience intolerable side effects [40, 41]. Aripiprazole is generally better tolerated but can still produce adverse reactions at higher dosages, and inadequate symptom improvement may lead to additional changes in treatment [41]. These dynamics underscore the individualized and iterative nature of pharmacological management of ASD [42]. Managing behavioral symptoms in ASD often involves ongoing adjustment based on individual response, tolerability, and clinical judgment [31]. Real-world evidence on how these medications are used may help complement clinical decision-making by providing additional context for treatment adjustments in routine practice.

The study has limitations. First, it primarily included individuals with commercial insurance, which may limit the generalizability of the findings to populations with different or no coverage. Second, ASD diagnoses were based on ICD-9/ICD-10 codes without direct clinical verification, and investigators lacked the data necessary to validate those diagnoses. Third, the clinical rationale behind prescribing SGAs or SSRIs is unknown, limiting the ability to interpret drivers of observed prescribing and switching profiles. This limitation stems from the nature of claims data, which primarily captures healthcare transactions between providers and payers for reimbursement purposes. As such, the investigators did not have access to physicians’ notes or other clinical documentation that could provide insight into decision-making processes, patient symptom profiles, or considerations guiding treatment choices. Fourth, the COVID-19 pandemic may have affected the psychotropic medication access, and subsequently, switching patterns due to behavioral services access and providers not being able to monitor individuals to determine safety for those medications [43]. This might have influenced prescribing changes during the study period, which we did not account for in this analysis. Further studies are needed to understand the COVID-19 impact on psychotropic prescribing utilization in individuals with ASD. Additionally, further studies are needed to assess the long-term side effects of medications to treat ASD and their association with switching psychotropic medications.

Our findings highlight the importance of pharmacological intervention in individuals with ASD and the value of careful clinical consideration, particularly when prescribing SGAs. Individuals who were newly prescribed SGAs were more likely to switch classes than individuals prescribed SSRIs, with switching from aripiprazole increasing over time and those from risperidone declining. These changes may reflect variation in side effect profiles, efficacy, or shifts in clinical presentation. The variability observed in prescribing and medication switching underscores the importance of regular monitoring, individualized treatment planning, and the continued integration of behavioral interventions alongside psychotropic medications.

## Supplementary Information

Below is the link to the electronic supplementary material.

Supplementary Material 1

## Data Availability

All analysis codes are available in a GitHub repository: [https://github.com/polijaimesb/2024\_PsychotropicMedicationsSwitchingPatterns\_ASD](https:/github.com/polijaimesb/2024_PsychotropicMedicationsSwitchingPatterns_ASD). The data that support the findings of this study are available from IQVIA, but restrictions apply to the availability of these data, which were used under license for the current study, and so are not publicly available.

## Abbreviations

ASD: Autism spectrum disorder
SSRIs: Serotonin reuptake inhibitors
SGAs: Second-generation antipsychotics
ICD-9: International Statistical Classification of Diseases and Related Health Problems, Ninth Revision
ICD-10: International Statistical Classification of Diseases and Related Health Problems, Tenth Revision
