# Supplementary material for: Utilization of psychotropic medications in individuals with autism spectrum disorder
Source: BMC Psychiatry. 2025 Dec 23;26:72. doi: 10.1186/s12888-025-07714-2 (PMC12836897; doi:10.1186/s12888-025-07714-2)
Supplement: Supplementary file 1 — Supplementary Material 1 [file 12888_2025_7714_MOESM1_ESM.docx]

**Online Supplementary Material**

All analysis codes are available in a GitHub repository: <https://github.com/polijaimesb/2024_PharmacotherapyPatternsForChallengingBehavior_ASD.git>

**Supplementary Material Table S1.** Demographic characteristics and number of individuals by calendar years between 2012 and 2021 for the individuals who did and did not receive any psychotropic medication

| Characteristics | No. of participants (%) | | | | |
| --- | --- | --- | --- | --- | --- |
|  | No medications | All | Only SGAs | Only SSRIs | Both   SGAs and SSRIs |
|  | n = 31,179 | (n = 15,976)^b^ | (n = 2,662)^d^ | (n = 9,387)^e^ | (n = 3,927)^c^ |
| Sex |  |  |  |  |  |
| Female | 6,978 (22.4) | 4,171 (26.10) | 506 (19.00) | 2,725 (29.00) | 940 (23.90) |
| Male | 24,200 (77.6) | 11,805 (73.90) | 2,156 (81.00) | 6,662 (71.00) | 2,987 (76.10) |
| Age at first ASD diagnosis |  |  |  |  |  |
| 2 to 5 | 11,832 (37.9) | 1,136 (7.10) | 280 (10.50) | 550 (5.90) | 306 (7.80) |
| 6 to 11 | 10,021 (32.1) | 5,033 (31.50) | 908 (34.10) | 2,770 (29.50) | 1,355 (34.50) |
| 12 to 17 | 5,836 (18.7) | 6,028 (37.70) | 847 (31.80) | 3,705 (39.50) | 1,476 (37.60) |
| 18 to 26 | 3,490 (11.2) | 3,779 (23.70) | 627 (23.60) | 2,362 (25.20) | 790 (20.10) |
| Calendar year |  |  |  |  |  |
| 2012 | 2,731 (8.76) | 1,707 | 507 (29.70) | 924 (54.13) | 276 (16.17) |
| 2013 | 3,326 (10.67) | 2,292 | 610 (26.61) | 1,313 (57.29) | 369 (16.10) |
| 2014 | 4,356 (13.97) | 2,597 | 599 (23.07) | 1,577 (60.72) | 421 (16.21) |
| 2015 | 5,720 (18.35) | 2,971 | 699 (23.53) | 1,797 (60.48) | 475 (15.99) |
| 2016 | 7,929 (25.43) | 3,619 | 795 (21.97) | 2,313 (63.91) | 511 (14.12) |
| 2017 | 11,253 (36.09) | 4,619 | 953 (20.63) | 2,998 (64.91) | 668 (14.46) |
| 2018 | 15,049 (48.27) | 5,943 | 1,286 (21.64) | 3,638 (61.21) | 1,019 (17.15) |
| 2019 | 19,514 (62.59) | 7,469 | 1,541 (20.63) | 4,678 (62.63) | 1,250 (16.74) |
| 2020 | 16,789 (53.85) | 6,838 | 1,261 (18.44) | 4,518 (66.07) | 1,059 (15.49) |
| 2021 | 16,539 (53.05) | 6,970 | 1,217 (17.46) | 4,678 (67.12) | 1,075 (15.42) |

Abbreviations: ASD, autism spectrum disorder. SSRIs, selective serotonin reuptake inhibitors. SGAs, second-generation antipsychotics.

^a^ The estimations of each demographic variable are calculated from the individuals who received a prescription of any of the two SGA medications (aripiprazole and risperidone) and SSRIs. Individuals included in the estimations can also receive medications different from those mentioned. ^b^ “All” is the total estimation of individuals with at least one prescription of SSRIs or SGA medications (limited to aripiprazole and risperidone).

^c^ “Both SGAs and SSRIs” indicates the individuals receiving the two drug classifications in the same year without verification of concurrency.

^d^ “Only SGAs” are the individuals who received aripiprazole or risperidone, the two SGAs selected.

^e^ “Only SSRIs” are the individuals who received only medications from the specified category, including fluoxetine, fluvoxamine, sertraline, citalopram, escitalopram, and paroxetine

**Supplementary Material Figure S2.** Annual trends and changes in the utilization of medications*


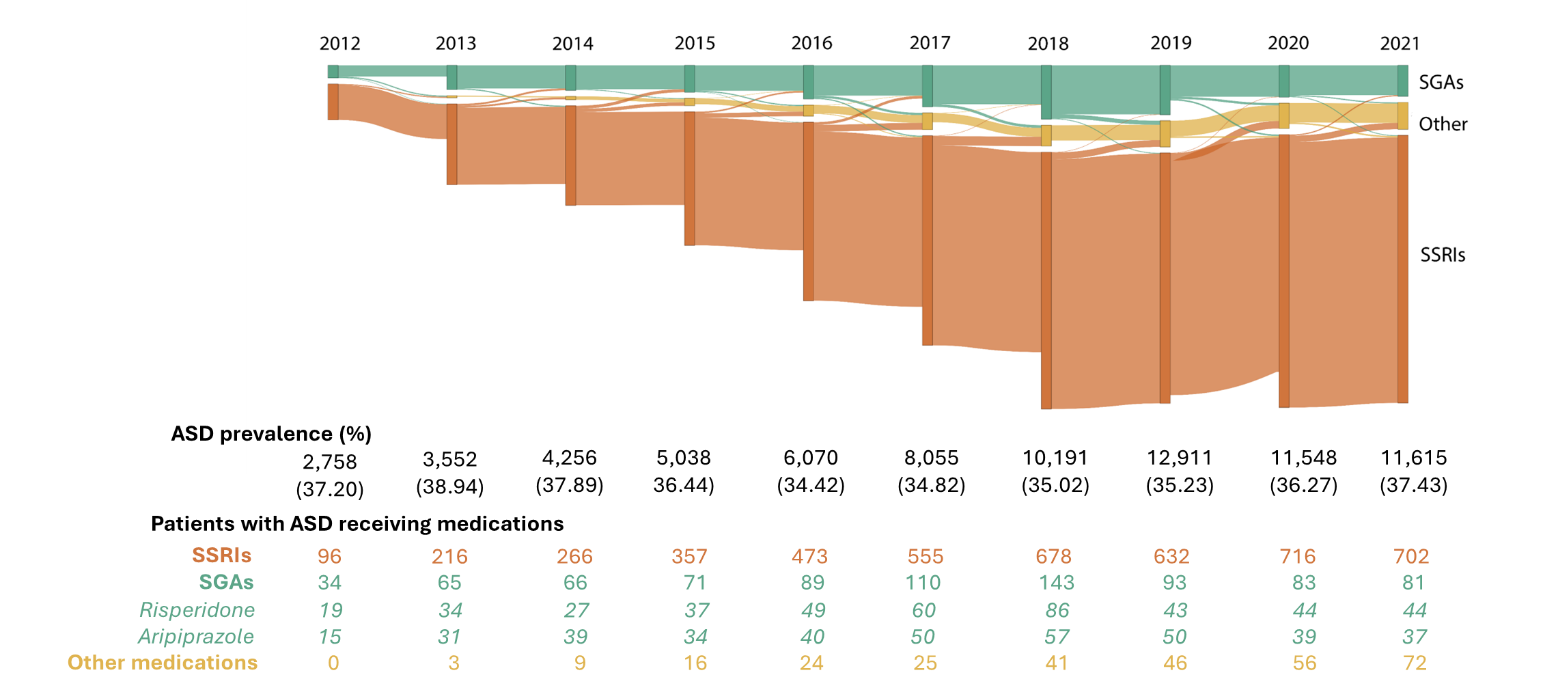


*Count of individuals in the bottom was stratified by SGAs included in the manuscript.

**Supplementary Material Figure S3.** Monthly trends and changes in the utilization of medications – 2019*


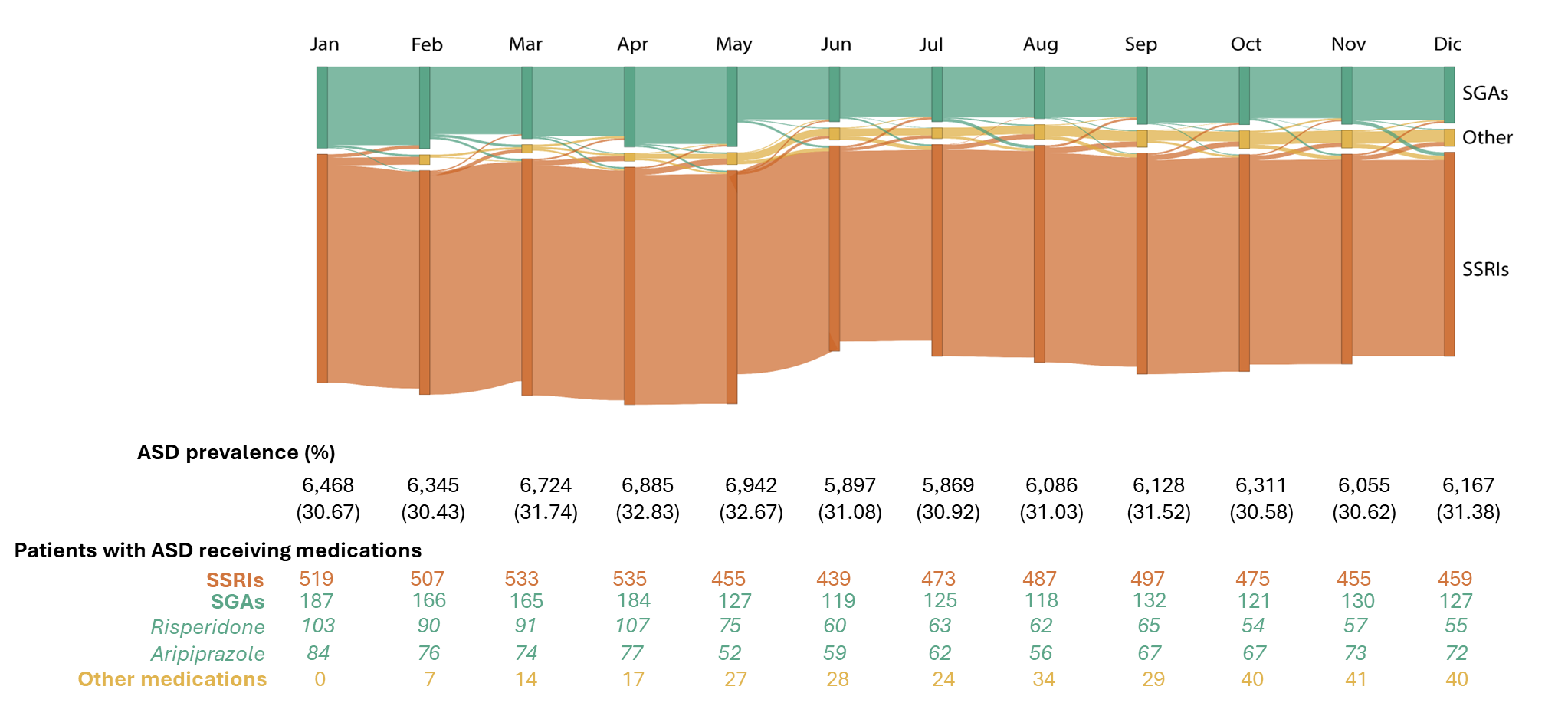


*Count of individuals in the bottom was stratified by SGAs included in the manuscript.
